# Rainfall-induced stability of unsaturated tropical slopes in Pagar Alam, South Sumatra, Indonesia: Effects of slope gradient and soil layering

**DOI:** 10.1371/journal.pone.0354028

**Published:** 2026-08-13

**Authors:** Madelestin Melhan, Eriko Dewangga, Alfrendo Satyanaga, Wiwik Rahayu, Abdul Halim Hamdany, Erly Bahsan, Glenn Adriel Adiguna, Martin Wijaya, Nurly Gofar, Herdian Gumay

**Affiliations:** 1 Department of Civil and Environmental Engineering, Faculty of Engineering, Universitas Indonesia, Depok, Jawa Barat, Indonesia; 2 Department of Civil Engineering and Environmental Engineering, School of Engineering and Digital Sciences, Nazarbayev University, Astana, Kazakhstan; 3 Department of Civil Engineering, Faculty of Engineering, Universitas Katolik Parahyangan, West Java, Indonesia; 4 Department of Civil Engineering, Postgraduate program, Universitas Bina Darma, Palembang, Indonesia; 5 Department of Civil Engineering, Faculty of Engineering, Universitas Hasanuddin, Gowa, Indonesia; Ardakan University, IRAN, ISLAMIC REPUBLIC OF

## Abstract

Rainfall-induced slope failure is a recurring geohazard in tropical regions, where intense and prolonged precipitation promotes infiltration, reduces matric suction, and increases pore-water pressure within near-surface soils. This study investigates the effects of slope gradient and soil layering on the stability of unsaturated natural slopes in Pagar Alam, South Sumatra, Indonesia, using soil properties obtained from laboratory testing. Three representative slope geometries derived from terrain data were analyzed under both homogeneous and layered subsurface conditions to assess the combined influence of topography and stratification. Transient rainfall infiltration was incorporated into a numerical framework, and stability was assessed using the Morgenstern-Price limit equilibrium method in GeoStudio. The results indicate that the factor of safety decreases rapidly due to rainfall infiltration. The greater the slope angle, the greater the reduction in the factor of safety. Layered slopes generally produced lower factors of safety than their homogeneous conditions, demonstrating that stratification can reduce slope stability even when the overall failure pattern remains similar. The results further suggest that hydraulic and mechanical contrasts between soil layers influence pore-water-pressure evolution and the distribution of shear resistance during rainfall. These findings highlight the importance of incorporating unsaturated soil behavior and realistic subsurface stratification into slope-stability assessment in tropical environments. The study provides a hydro-geologically relevant basis for improving landslide susceptibility evaluation and supports the development of site-specific monitoring and mitigation strategies for rainfall-prone slopes in Indonesia.

## Introduction

Slope instability is one of the most critical geohazards in tropical regions, where high-intensity and long-duration rainfall frequently trigger shallow and deep-seated landslides. In Indonesia, rainfall-induced failures are particularly common in mountainous areas characterized by steep topography, deeply weathered soil profiles, and strong seasonal precipitation [1–3]. Pagar Alam, South Sumatra, represents such conditions and is highly susceptible to slope failure due to its steep natural terrain and high annual rainfall exceeding 3000 mm [1,4,5]. The interaction between hydrological processes and soil mechanical behavior makes slope stability in this region both critical and challenging to assess.

The destabilizing effect of rainfall is governed primarily by hydromechanical changes within unsaturated soils. Above the groundwater table, soils are typically in an unsaturated state, where matric suction contributes to effective stress and enhances shear strength [6–8]. However, rainfall infiltration reduces matric suction and may generate positive pore-water pressure, especially when infiltration exceeds drainage capacity or where hydraulic contrasts exist within the soil profile. This process reduces available shear resistance and may trigger failure when the balance between resisting and driving forces becomes unfavorable. [9–11]. The relationship between suction and water content of soil can be defined using the soil-water characteristic curve (SWCC), which governs hydraulic conductivity behavior [12,13]. Previous studies have shown that rainfall intensity and duration strongly influence slope response: short-duration, high-intensity rainfall tends to induce shallow failure; whereas prolonged rainfall promotes deeper instability through pore-pressure buildup [2,3,14].

Among the factors affecting rainfall-induced instability, slope gradient is one of the most influential geometric parameters. Steeper slopes generally produce larger driving forces and higher mobilized shear stress along potential slip surfaces, thereby reducing the margin of safety. However, topographic steepness alone is insufficient to explain the behavior of natural slopes. In tropical environments, soil profiles are rarely homogeneous and commonly consist of layered materials with varying hydraulic and mechanical properties [15–17]. Soil layering introduces variability that influences infiltration behavior and pore-water distribution, which promotes failure along weaker interfaces.

Despite this, many conventional analyses still assume homogeneous conditions, potentially leading to inaccurate predictions. This issue is particularly relevant in Indonesia, where rainfall-triggered landslides often occur in steep weathered slopes, yet engineering assessments still frequently rely on homogeneous models for simplicity. Such simplifications may overestimate slope stability and underestimate the influence of hydromechanical heterogeneity [2,3,10,14].

Accordingly, this study evaluates the influence of slope gradient and soil layering on rainfall-induced slope stability in Pagar Alam, South Sumatra, Indonesia, using representative local soil properties and an unsaturated-soil numerical framework. Three slope geometries were derived from terrain data and analyzed under both homogeneous and layered conditions. The objective is to examine how slope geometry and subsurface stratification interact to control the evolution of pore-water pressure and the factor of safety during rainfall infiltration. The study provides a mechanistic numerical assessment rather than a fully calibrated reconstruction of a specific landslide event; nevertheless, it provides insight into how realistic stratification and unsaturated behavior should be incorporated into slope analysis and landslide-risk mitigation in tropical environments.

## Methodology

### Study framework

This study evaluates rainfall-induced slope stability under unsaturated conditions using representative natural slopes from Pagar Alam, South Sumatra, Indonesia. Located near the mountain range of Bukit Barisan, the region is widely known for slope failure cases, mainly caused by water infiltration [5]. To comprehend influence of the hydraulic condition on the slope behavior, the analysis was carried out within a numerical framework that links transient infiltration behavior and limit-equilibrium slope-stability assessment. The study focuses on two governing variables: slope gradient and soil layering. To isolate their influence, three slope geometries were analyzed under two subsurface scenarios, namely homogeneous and layered conditions, while the rainfall loading procedure and boundary assumptions were kept consistent among all models.

The slope stability analysis was performed using SEEP/W and SLOPE/W modules in a coupled workflow. Initially, SEEP/W was used to simulate the groundwater conditions, with the water table set at 12–13 m below the surface, following the slope geometry to approximate natural subsurface conditions. The groundwater table depth in Pagar Alam generally ranges from 3 to 12 meters below ground surface, based on the depth of community wells [18]. Boundary conditions included constant water pressure along the base and lateral boundaries, while no-flow conditions were applied on the left and right of the model boundaries before groundwater establishment. Surface infiltration representing rainfall was applied at the top of the slope to simulate recharge. Once the groundwater distribution stabilized, the results from SEEP/W were imported into SLOPE/W for the slope stability analysis. The critical slip surface was determined using the Morgenstern-Price method, which divides the potential sliding mass into vertical slices, calculates interslice forces, and iteratively balances moments and forces to determine the factor of safety. This method allows a rigorous evaluation of slope stability under the influence of the prevailing pore-water pressures obtained from the seepage analysis.

The work is intended as a scenario-based mechanistic study using local soil data rather than a complete back-analysis of a specific landslide event. Therefore, based on the variations of slope gradient and soil stratification, the results are interpreted in terms of comparative slope response, factor-of-safety reduction, and hydromechanical trends during rainfall infiltration.

### Slope geometry

**Fig 1:**
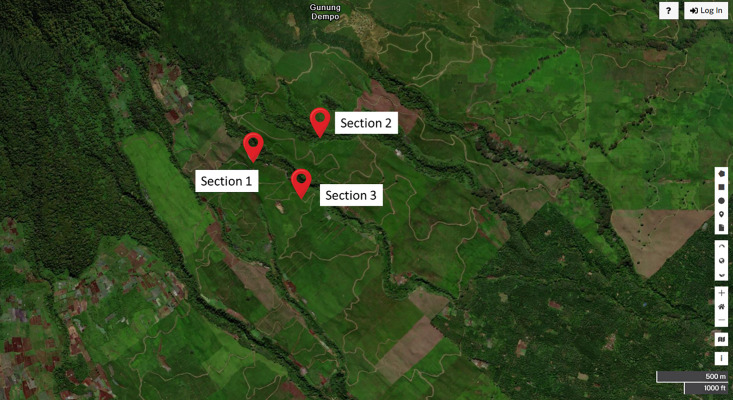
Pagar Alam’s contour map (NASA Earth Observatory (public domain)).

**Fig 2:**
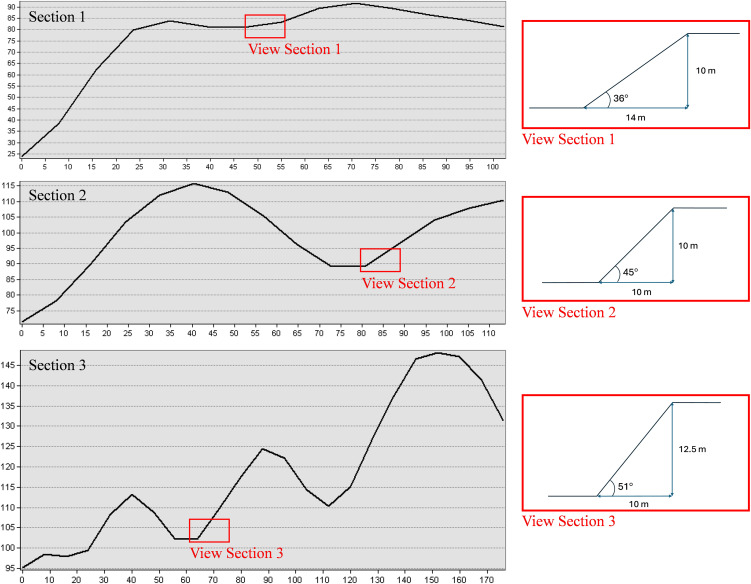
Section variation of Pagar Alam contour.

Three representative slope sections were selected from terrain profiles generated using ArcGIS processing. The map was extracted from NASA Earth Observatory (public domain) which does not require permission from the source. These sections were chosen to represent different slope gradients observed in Pagar Alam area rather than to represent statistically random samples. The term representative sections are used intentionally because the purpose of the selection was to capture geometric variation rather than to conduct statistical random sampling. The main sections are identified in Fig 1 as Section 1 (Longitude 103° 9’0.21” E, Latitude 4° 3’0.25” S), Section 2 (Longitude 103° 9’15.30” E, Latitude 4° 2’54.32” S), and Section 3 (Longitude 103° 9’10.64” E, Latitude 4° 3’8.64” S). The selected sections were extracted and simplified into two-dimensional slope models for numerical analysis in Fig 2.

Each section was analyzed using two material configurations: [1] a homogeneous slope, in which the entire slope body was assigned a single representative soil type based on laboratory-tested properties, and [2] a layered slope, in which the slope body was divided into three layers representing a weathered upper zone, an intermediate in-situ soil layer, and a denser lower layer. This modelling strategy allows the influence of stratification to be assessed while preserving identical slope geometry for direct comparison.

### Soil characterization and layered-profile assumptions

The middle layer, referred to as Soil B, represents the principal in-situ soil condition and is based on laboratory data obtained from soil samples collected in Pagar Alam in March 2024. Two additional materials were introduced to represent generalized vertical variation commonly observed in tropical weathered profiles. Soil A corresponds to a more weathered and weaker near-surface layer, whereas Soil C represents a denser and less permeable lower layer. Soil A and Soil C were not derived from in-situ sampling, but their parameters were established using a combination of published ranges for tropical soils, regional geotechnical experience, and engineering judgment to ensure realistic stratification. Soil A and Soil C are therefore interpreted as idealized but geotechnically reasonable materials intended to simulate realistic stratification; they do not represent direct layer-by-layer measurement from a fully characterized borehole profile.

**Fig 3:**
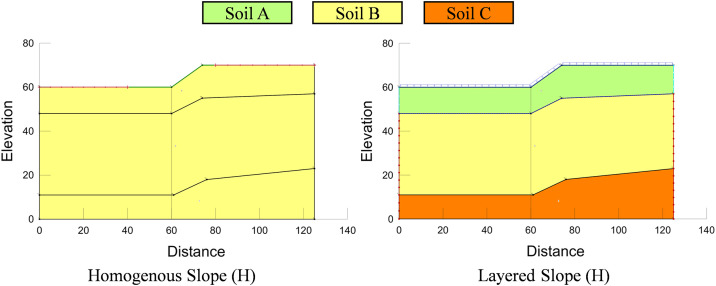
Section variation for simulation.

The thickness and arrangement of soil variation adopted in this study were conceptual and intended for idealized modeling rather than direct replication of a fully characterized field profile (Fig 3). In the layered configuration (*L*), Soil A and Soil C were each assigned to a thickness of 12 meters with Soil B in the middle, imitating the typical soil profile from the site boreholes results. On the other hand, the homogeneous configuration (*H*) assumed a single equivalent soil profile of Soil B for the entire slope.

All models were analyzed under consistent boundary conditions. The initial groundwater table was assigned at approximately 12–13 meters below the surface, following the slope profile to represent natural subsurface conditions. In order to establish a stable hydrostatic state, the lateral boundaries were applied as no flow rate conditions at this depth on left and right model boundaries. Once, the groundwater table was initialized, a constant water-pressure boundaries were assigned along the lateral limits. Rainfall was simulated as surface infiltration, enabling transient pore-water pressure development within the slope.

**Fig 4:**
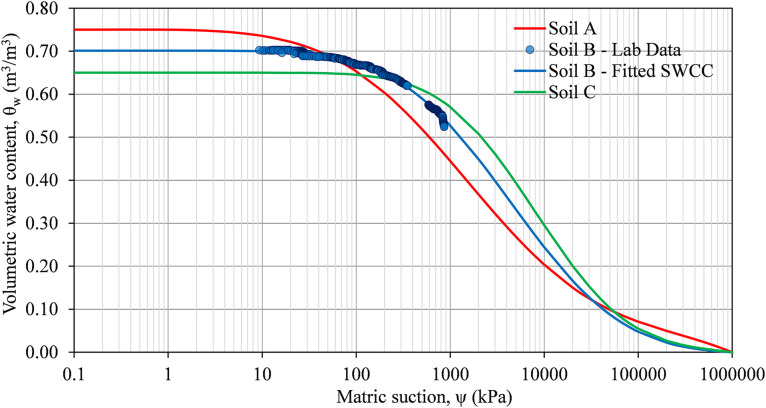
SWCC of Soil A, B, and C.

**Fig 5:**
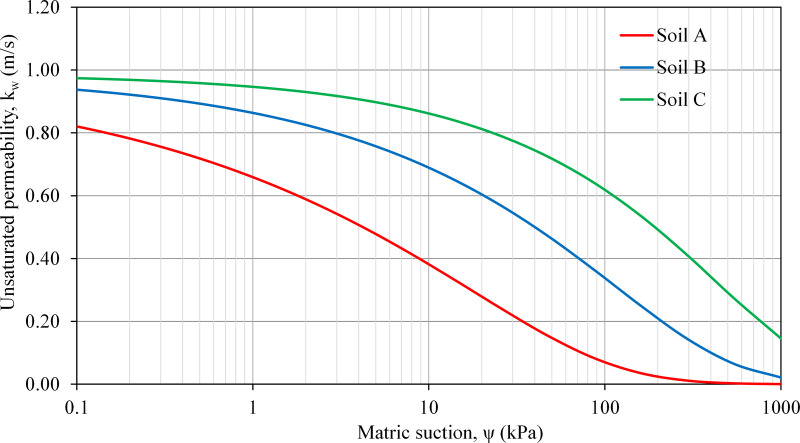
Unsaturated permeability function of Soil A, B, and C.

The primary input parameters include natural water content, specific gravity, density, friction angle, cohesion, elastic modulus, unit weight, and saturated hydraulic conductivity. Hydraulic behavior under unsaturated conditions was incorporated through the soil-water characteristic curve (SWCC) and unsaturated permeability function, obtained from laboratory measurements. The use of an SWCC-based hydraulic model is necessary because the rate of wetting-front advance in unsaturated soils depends not only on saturated conductivity but also on the changing relationship between suction and water content. The soil parameters used in this study are presented in Table 1, with SWCC shown in Fig 4, and the unsaturated permeability function in Fig 5.

**Table 1:** Soil parameter.

Parameter	Soil A	Soil B*	Soil C
Water content natural (%)	50	45	35
Specific gravity	2.60	2.60	2.62
Density (g/cm^3^)	1.60	1.72	1.80
Friction angle (°)	25	27	30
Cohesion (kPa)	20	32	50
Elastic modulus (kPa)	6,000	10,000	20,000
Unit weight (kN/m^3^)	15.50	16.9	18.00
Hydraulic conductivity (m/s)	1.00E-05	1.02E-06	1.00E-07
**Best Fitted Parameter [19]**			
Saturated volumetric water content,$\theta_{s}$	0.750	0.701	0.650
Residual volumetric water content,$\theta_{r}$	0.050	0.002	0.010
Soil air-entry value, $\psi_{a}$ (kPa)	0.8	1.386	3.0
Matric suction at inflection point, $\psi_{m}$ (kPa)	1500	4352.95	8000
Geometric standard deviation,*σ*	2.5	2.17	1.80
Matric suction at residual volumetric water content, $\psi_{r}$ (kPa)	1,000,000	2,934,175	5,000,000

*Actual data from laboratory test with Pagar Alam’s soil

The equation used for best fitting the SWCC is as follows [19]:


$$\begin{array}{c} \theta_{w}=\left(\text{1}-\frac{\text{ln}\left(\text{1}+\frac{\psi }{\psi_{r}}\right)}{\text{ln}\left(\text{1}+\frac{{\text{1}\text{0}}^{\text{6}}}{\psi_{r}}\right)}\right)\left[\theta_{r}+\left\{\left(\theta_{s}-\theta_{r}\right)\left(\text{1}-(\beta )erfc\left(\frac{\text{ln}\left(\frac{\psi_{a}-\psi }{\psi_{a}-\psi_{m}}\right)}{\sigma }\right)\right)\right\}\right] \end{array}$$ (1)

Whereas β = 0 when ψ ≤ $\psi_{a}$; β = 1 when ψ> $\psi_{a}$.

### Shear-strength representation under unsaturated conditions

**Fig 6:**
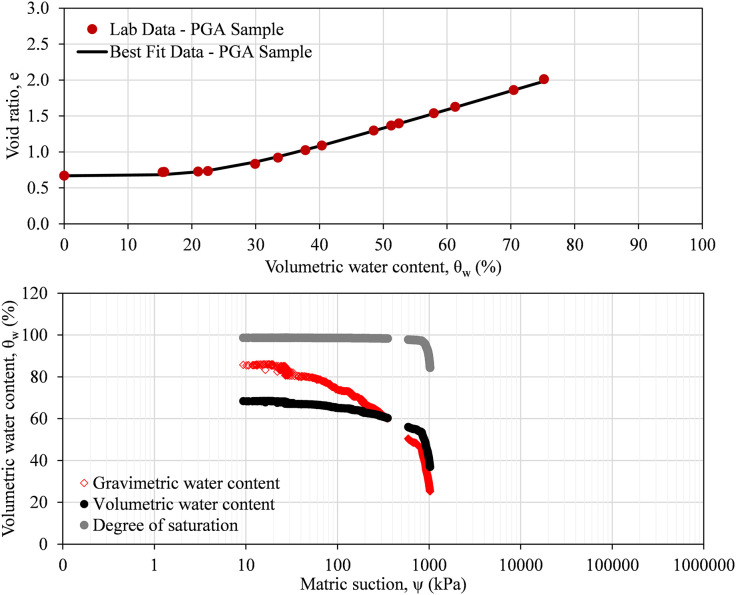
Shrinkage curve of Pagar Alam soil.

The SWCC of Soil B (Fig 4), natural Pagar Alam’s soil, shows that at low suction levels (approximately 10–50 kPa), the soil is nearly saturated, with a high degree of saturation and relatively high water content, indicating that permeability is also high, and water can flow easily through the pore spaces. The void ratio versus water content relationship in Fig 6 further indicates that increasing water content leads to an increase in void ratio, suggesting that the soil structure becomes looser or undergoes slight swelling upon wetting. This behavior is typical of fine-grained soils and reflects hydro-mechanical coupling, where an increase in moisture reduces effective stress and weakens interparticle bonding [20,21].

These findings align with the expected response of unsaturated fine-grained soils under rainfall conditions. The high initial water retention and suction-controlled strength provide apparent stability under dry conditions, while the progressive loss of suction during infiltration leads to a rapid reduction in shear strength [22]. This mechanism is widely recognized in residual and colluvial soils in humid regions, where rainfall-induced failures are often triggered by the collapse of matric suction rather than a sudden increase in pore-water pressure alone. Therefore, slope stability in Pagar Alam is strongly governed by the interaction between unsaturated soil behavior, rainfall infiltration, and soil layering.

The mechanical effect of unsaturation was considered through the contribution of matric suction to shear strength. Under rainfall infiltration, the magnitude of matric suction decreases, thereby reducing the suction-related component of shear resistance. This mechanism is fundamental to rainfall-induced slope instability in initially unsaturated slopes and provides the conceptual basis for linking transient seepage behavior to factor-of-safety reduction [23].

### Rainfall loading

**Fig 7:**
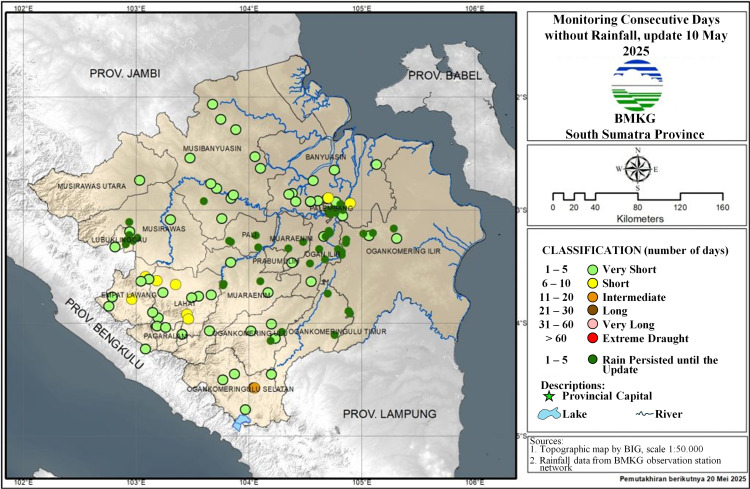
Number of days without constitutive rain [24].

Rainfall input was defined using monthly precipitation and rainy-day data for Pagar Alam from the Central Bureau of Statistics of Pagar Alam City (Indonesia Central Bureau of Statistics or BPS) for the year 2021, as summarized in Table 2. The year 2021 was selected because it represents the most recent and complete dataset publicly available from the official BPS of Pagar Alam city at the time of analysis. More recent data is not accessible in the official database. For numerical consistency, rainfall was represented as an infiltration boundary condition based on a selected characteristic intensity derived from the available dataset, rather than an event-based storm hyetograph. According to the Indonesian Meteorological, Climatological, and Geophysical Agency (BMKG), the number of consecutive dry days in Pagar Alam is very short, typically ranging from one to five days, as shown in Fig 7. This indicates that Pagar Alam experiences frequent rainfall occurrences. Therefore, the rainfall was assumed to be uniformly distributed across rainy days, allowing a representative rainfall intensity to be calculated. Although the highest rainfall occurred in March, variation in the number of rainy days resulted in the highest calculated rainfall intensity in April, i.e., 2.58 × 10^−4^ mm/sec. The rainfall duration considered in this study is 14 days, which follows the BMKG classification of an intermediate number of rainy days (11–20 days). This duration was selected to represent a typical condition within this category for the rainfall event analysis.

**Table 2:** Rainfall intensity of Pagar Alam in 2021 based on BPS [25].

Month	Rainfall (mm)	Rainy days	Intensity	
			(mm/s)	(m/s)
January	214	17	1.46E-04	1.46E-07
February	214	19	1.30E-04	1.30E-07
March	405	26	1.80E-04	1.80E-07
April	356	16	2.58E-04	2.58E-07
May	305	17	2.08E-04	2.08E-07
June	167	18	1.07E-04	1.07E-07
July	83	12	8.01E-05	8.01E-08
August	97	11	1.02E-04	1.02E-07
September	279	18	1.79E-04	1.79E-07
October	272	20	1.57E-04	1.57E-07
November	247	17	1.68E-04	1.68E-07
December	55	27	2.36E-05	2.36E-08

### Stability analysis

Slope stability was evaluated using the Morgenstern-Price method [26], which satisfies both force and moment equilibrium. The method has been widely used in geotechnical engineering for rigorous capability to satisfy force and moment equilibrium in the analysis. The term Factor of Safety (FS) is applied to define the ratio of resisting driving forces [27]. The FS was monitored over time as rainfall infiltration progressed. Critical slip surfaces were identified automatically within the search domain for each analysis stage using entry-exit method.

### Comparative analysis strategy

The analysis was designed to compare: [1] the effect of slope gradient across the three representative sections, [2] the difference between homogeneous and layered conditions for each section, [3] the time-dependent change in FS during rainfall infiltration, and [4] the evolution of pore-water pressure profiles at selected times. This structure allows the relative influence of geometry and stratification to be assessed systematically. The purpose is not only to identify which slope is least stable, but also to examine how soil layering modifies the hydraulic-mechanical response during rainfall.

## Results

**Fig 8:**
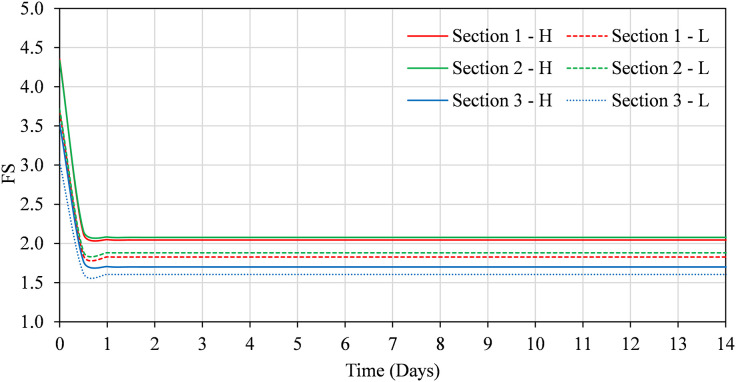
FS results throughout rainfall infiltration.

Rainfall infiltration caused a marked reduction in slope stability for all analyzed sections, with the FS declining sharply during the early stages of rainfall and gradually approaching a near-steady value as hydraulic conditions stabilized, as shown in Fig 8 and Table 3. The initial decrease in FS reflects the rapid rise of pore-water pressure within the infiltrated zone. This rapid response is consistent with the relatively high permeability of soils in Pagar Alam, which allows rainfall to infiltrate and flow quickly inside the soil pores and generate significant porewater pressure within the slope. The rapid build-up of porewater pressure lowers the shear strength of the soil, which becomes the reason for the FS reduction. This highlights that the greatest decrease in stability occurs during the early infiltration phase.

**Table 3:** FS and the reduction to the initial condition.

Section	Time						
	Initial	12 hours		1 day		14 days	
	FS	FS	Reduction (%)	FS	Reduction (%)	FS	Reduction (%)
1 – H	4.35	2.12	51.20	2.05	52.92	2.04	53.04
1 – L	3.68	1.85	49.83	1.83	50.34	1.83	50.38
2 – H	4.33	2.16	50.13	2.08	51.92	2.08	52.05
2 – L	3.72	1.90	48.96	1.88	49.38	1.88	49.41
3 – H	3.53	1.77	49.81	1.70	51.73	1.70	51.87
3 – L	3.04	1.62	46.61	1.61	47.10	1.61	47.14

**Fig 9:**
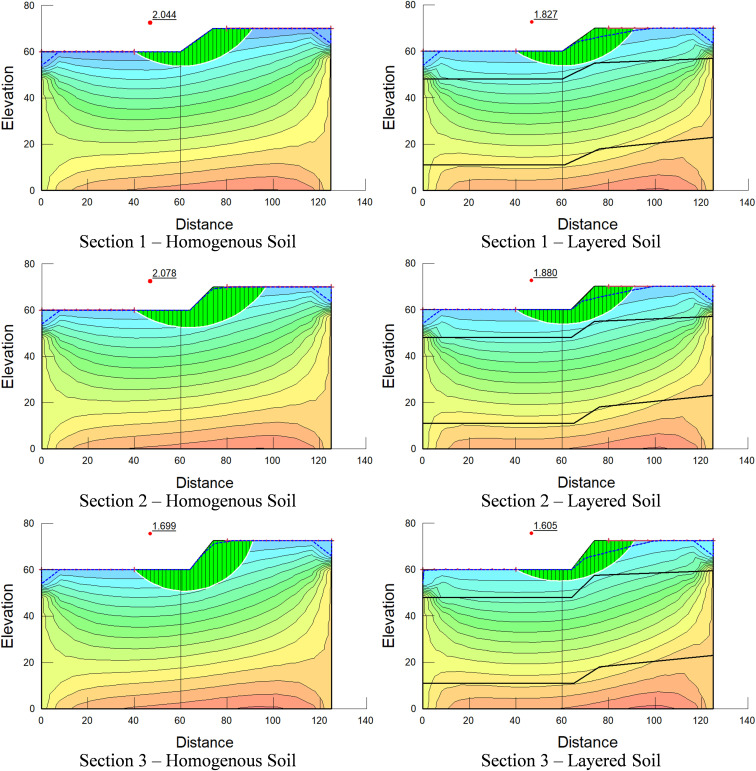
Critical slip surface results.

The results also show that slope gradient exerts a strong control on stability. The steeper sections consistently produced lower factors of safety than the gentler sections, confirming that higher driving forces increase susceptibility to rainfall-induced instability. Among the analyzed cases, the steepest section exhibited the lowest final factor of safety, indicating that slope geometry remains the dominant first-order control on overall stability [15]. When homogeneous and layered models are compared for the same slope geometry, the layered slopes generally produce lower FS. This indicates that stratification can reduce slope stability even when the overall failure mechanism remains similar. In all scenarios, circular slip surfaces were observed (Fig 9), suggesting that soil layering and hydraulic contrasts influence the magnitude and timing of instability rather than fundamentally altering the failure mode.

**Fig 10:**
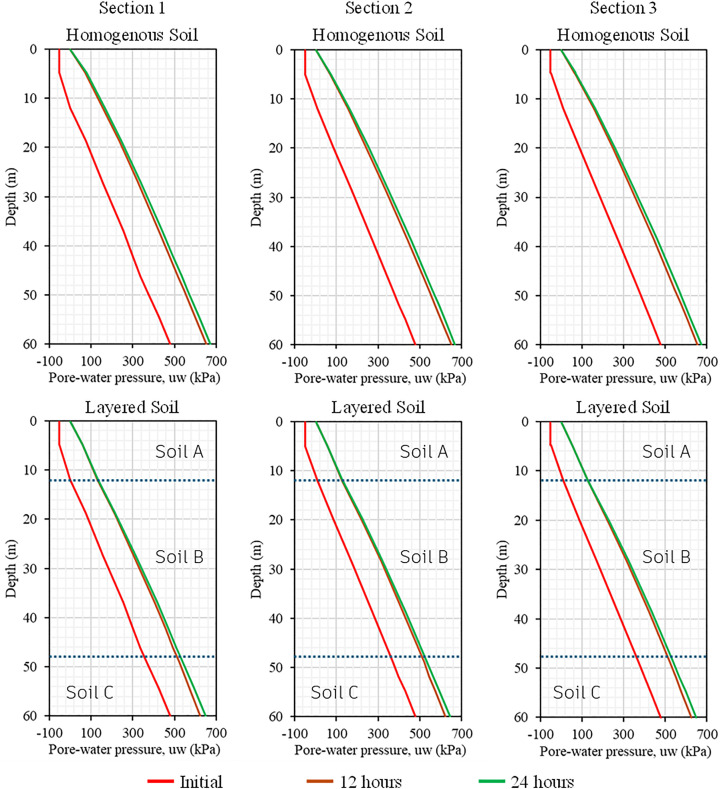
Pore-water pressure at initial condition (left), 12 hours (middle), and 1 day (right).

Fig 10 illustrates the effect of stratification on pore-water pressure profiles from 0, 12, and 24 hours. The values from the subsequent durations are not shown due to the insignificant changes. During these 14 days, layered slopes consistently exhibited lower pore-water pressures compared to homogeneous slopes, indicating that contrasts in hydraulic and mechanical properties between layers can redistribute flow paths and shear resistance, thereby modifying the evolution of slope stability during rainfall. However, the trend of the pore-water pressure changes is similar between homogenous and layered models, in which a significant increase in pore-water pressure occurred within the first 12 hours. Subsequently, a small increase was observed among the sections under 24 hours, with negligible changes taking place afterward. Overall, these results highlight the combined influence of slope gradient, soil properties, and stratification on rainfall-induced instability in tropical slopes.

## Discussion

The numerical results confirm that rainfall-induced slope instability in Pagar Alam cannot be interpreted solely as a geometric problem. Although steeper slopes exhibit a greater reduction in safety because of larger driving forces, the infiltration response and internal hydraulic structure of the slope are also critical [28,29]. The simulations depicted a rapid reduction of matric suction and an increase in porewater pressure due to rainfall infiltration. In hydrogeologic terms, the slope behaves as a shallow variably saturated flow system in which rainfall recharge, storage change, and conductivity contrasts jointly determine the pressure response that drives mechanical weakening.

The results are consistent with the characteristics of fine-grained soils, commonly found in tropical regions such as Pagar Alam. These soils generally have a high capacity to retain water and exhibit significant changes in mechanical behavior with variations in moisture content. The observed response indicates moderate plasticity and a strong influence of capillary effects, which are typical features of soils with a well-developed fine pore structure. These imply that the soil is highly sensitive to rainfall infiltration [30]. During the early stages of rainfall, the slope remains relatively stable due to matric suction. However, as infiltration continues, suction decreases, and permeability allows water to move into the soil profile, weakening the material [31,32].

Soil layering further influences stability, although it does not alter the failure mechanism. The layered slopes consistently produced lower factors of safety than the homogeneous slopes, even where the plotted pore-water-pressure profile appeared lower at selected depths. This outcome is plausible because global slope stability does not depend exclusively on the magnitude of pore-water pressure at one profile location; rather, it depends on the combined distribution of shear strength, hydraulic gradients, and potential failure-path geometry across the slope mass [33]. A layered model may therefore be less stable if a weaker or less favorable interface controls the critical slip surface, even when average pore-water pressure at a selected section is not higher. Accordingly, ignoring stratification can lead to overestimation of stability and underestimation of landslide risk, emphasizing the importance of incorporating realistic layered soil profiles in tropical slope assessments.

From a hydrogeologic perspective, the results demonstrate that slopes behave as shallow, variably saturated flow system in which rainfall recharge, storage changes, and permeability contrasts with the control pressure response that drives mechanical weakening [34]. The results also have practical implications for landslide-risk evaluation in tropical terrains. Assessments based only on homogeneous soil properties may overestimate slope stability and underestimate the effect of local hydraulic heterogeneity. In data-limited regions, a conceptual layered model informed by field observation and laboratory testing may therefore provide a more defensible basis for hazard screening than a fully homogeneous approximation. For hydro-geologically sensitive slopes, targeted monitoring of rainfall, suction, shallow groundwater response, and near-surface moisture conditions would be especially valuable for validating such models and improving early warning capability.

This study is based on numerical modelling using representative soil parameters and idealized layered profiles. Although the results provide useful mechanistic insight into slope behavior, they do not fully capture site-specific variability in weathering structure, hydraulic anisotropy, preferential flow, antecedent moisture, or rainfall intensity fluctuation. Additionally, the rainfall loading was based on representative intensity derived from monthly data rather than a fully resolved storm hyetograph.

## Conclusions and recommendations

This study examined the combined effects of slope gradient and soil layering on rainfall-induced instability of unsaturated slopes in Pagar Alam, South Sumatra. The numerical results demonstrate that slope stability decreases rapidly after rainfall infiltration begins and that steeper slopes are more vulnerable to instability because they mobilize larger driving forces. However, slope gradient alone is insufficient to fully assess stability, as soil layering influences infiltration response, matric suction loss, and spatial distribution of shear resistance. The analysis also shows that layered slopes generally produce lower factors of safety than equivalent homogeneous slopes, indicating that stratification can reduce slope stability even when the overall failure mechanism remains broadly similar.

These findings highlight the importance of incorporating hydro-stratigraphic representation and unsaturated-soil behavior should be considered into slope-stability assessments in tropical environments. For rainfall-prone slopes in Indonesia, mechanistic models that consider both topography and subsurface heterogeneity can provide a more defensible basis for susceptibility evaluation, monitoring design, and mitigation planning.

Future work should include field verification of the assumed layered profile, direct monitoring of pore-water pressure and suction during rainfall events, and calibration of the model using site-specific groundwater observations or landslide case histories. A more detailed hydrogeologic characterization, including the role of preferential seepage paths and seasonal groundwater dynamics, would further improve the realism and predictive value of the analysis. Further investigations are also recommended to assess the effectiveness of various slope stabilization and mitigation strategies under different soil layering scenarios.
